# Incentives and Reminders to Improve Long-term Medication Adherence (INMIND): Protocol for a Pilot Randomized Controlled Trial

**DOI:** 10.2196/42216

**Published:** 2022-10-31

**Authors:** Chad Stecher, Ishita Ghai, Lillian Lunkuse, Peter Wabukala, Mary Odiit, Agnes Nakanwagi, Sebastian Linnemayr

**Affiliations:** 1 College of Health Solutions Arizona State University Phoenix, AZ United States; 2 Pardee RAND Graduate School Santa Monica, CA United States; 3 Mildmay Uganda Kampala Uganda; 4 RAND Corporation Santa Monica, CA United States

**Keywords:** medication adherence, HIV, antiretroviral therapy, habit formation, routines, behavioral economics

## Abstract

**Background:**

Nonadherence to antiretroviral therapy (ART) among people living with HIV is a crucial barrier to attaining viral suppression globally. Existing behavioral interventions have successfully increased ART adherence, but typically show only short-term impact that dissipates after the interventions are withdrawn.

**Objective:**

This study aims to test the feasibility, acceptability, and preliminary efficacy of a novel intervention that uses SMS text messages and conditional incentives to support ART initiators in establishing pill-taking habits.

**Methods:**

A sample of 150 participants aged ≥18 years who have initiated ART in the preceding 3 months will be recruited from Mildmay Uganda in Kampala, Uganda. All (150/150, 100%) participants will be educated on the anchoring strategy and will choose an existing routine to pair with their daily ART adherence from a set of 3 suggested routines: getting dressed in the morning, eating breakfast, or eating dinner. Then, participants will be randomized to receive either usual care (control group: 50/150, 33.3%) or 1 of the 2 interventions delivered over 3 months: daily SMS text message reminders to follow their chosen anchoring plan (*messages* group; treatment group 1: 50/150, 33.3%) or daily SMS text messages and incentives conditional on taking their ART medication around the time of their chosen anchor (*incentives* group; treatment group 2: 50/150, 33.3%). Long-term ART adherence will be evaluated for 6 months after the intervention, and survey assessments will be conducted at baseline, 3 months, and 9 months. Outcomes include feasibility and acceptability measures and intervention efficacy outcomes defined by electronically measured mean medication adherence during the intervention and during the 6 months after the intervention, along with a measure of routine ART adherence based on taking medications around the time of participants’ anchor during the intervention and during the 6 months after intervention.

**Results:**

As of February 18, 2022, recruitment was completed. A total of 150 participants were recruited, and data collection is expected to end in December of 2022. Final results are expected to be submitted for publication by April 2023.

**Conclusions:**

This study is the first to use behavioral economics–based interventions in combination with the anchoring strategy to improve long-term ART adherence among treatment initiators. We hypothesize that the combination of SMS text message reminders and incentives will increase participants’ use of their anchoring strategy, and thus medication adherence will be better maintained after the intervention ends in our intervention groups relative to the control group that uses only the anchoring strategy. Results of this pilot study will help to refine this combined intervention approach for testing at scale and broaden our understanding of the habit formation process.

**Trial Registration:**

ClinicalTrials.gov NCT05131165; https://clinicaltrials.gov/ct2/show/NCT05131165

**International Registered Report Identifier (IRRID):**

DERR1-10.2196/42216

## Introduction

### Background

Antiretroviral therapy (ART) has transformed an HIV infection from a likely death sentence to a manageable chronic condition [1], but the efficacy of ART hinges on maintaining high (at least 80%-85%) mean medication adherence [2-4]. Globally, approximately 53% of people living with HIV have access to ART, but only 44% of people living with HIV are virally suppressed [5]. In sub-Saharan Africa, only one-fourth of people living with HIV are virally suppressed [5], which results in avoidable cases of drug resistance [6] and death [7]. Structural (eg, drug availability), social (eg, stigma), and economic (eg, distance to clinic and clinic fees) ART adherence barriers have been documented in the literature [8-10], but patient behavior has been identified as a key factor determining the lack of viral suppression [11]. Recent studies have shown that mean ART adherence ranges from 60% to 80% in Uganda, and only 30% to 60% of patients achieve 85% adherence [12-14]. Thus, novel behavioral interventions are needed to help establish and maintain high ART adherence habits among people living with HIV in Uganda.

**Figure 1:**
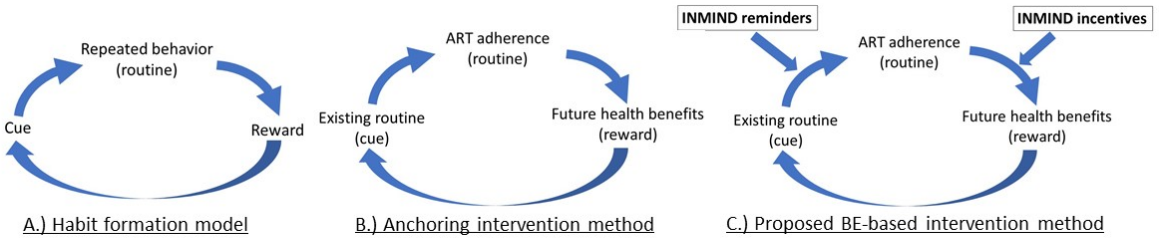
Depiction of the habit formation model by Duhigg [21] that underlies the anchoring intervention strategy, which we propose to enhance in Incentives and Reminders to Improve Long-term Medication Adherence (INMIND) through incentives and reminders to successfully routinize antiretroviral therapy (ART) adherence among patients with HIV. BE: behavioral economics.

Daily habits (or routines) are a commonly reported strategy for maintaining high medication adherence among patients who successfully manage chronic diseases [15,16], but forming new routines is often difficult for patients to do on their own. According to recent psychology research, it takes approximately 3 months of repeatedly performing a daily behavior in response to the same contextual cue [17-20], as outlined in the Habit Formation Model in Figure 1A [21], before the behavior becomes routinized. Once routinized, the cognitive processes that govern the behavior move to neurological systems that operate nonconsciously [22-25]. In addition, behaviors that are routinized no longer require high intrinsic motivation to be performed, and thus can enable even the most vulnerable ART treatment initiators, such as those with limited motivation, to maintain high long-term adherence.

A common intervention method for establishing new routines is to anchor the targeted behavior to an existing routine that acts as the contextual cue. For example, taking ART medication after brushing one’s teeth in the morning or after completing evening prayers. This method is often called anchoring (Figure 1B) and has been shown to be an effective intervention for promoting physical activity routines [26], improving dietary routines [27], and maintaining smoking cessation [28]. However, these existing studies typically enrolled participants with high intrinsic motivation for the targeted behavior [29-33], and therefore have little potential for real-life clinical situations where there is no extra support for individuals with low intrinsic motivation (as is typical for many patients in HIV care [34]). Moreover, less than half of the participants in these existing studies successfully used their anchor and maintained the desired behavior in the long term [35].

Behavioral economic theory demonstrates the need for ongoing support during the time it takes to complete the routinization process and provides proven intervention methods for delivering such support to enhance existing anchoring interventions. The behavioral economic biases of lack of salience of ART adherence (eg, over time, more pressing needs of daily life dominate one’s attention and focus) and present bias (eg, excessively undervaluing the future health benefits of one’s actions) help to explain why many people have trouble adhering to their healthy intentions [36,37]. Fortunately, behavioral economics also suggests 2 methods for countering these biases: (1) SMS text messages that can be used to reinforce the information provided at recruitment and to increase the salience of the anchoring routinization strategy and (2) small behavioral economics–based incentives that have successfully changed a range of health behaviors by countering present bias [38-41]. Therefore, this study will test whether incentives for linking daily pill taking to the timing of an existing routine behavior can establish and maintain high ART adherence routines in a feasible and scalable manner.

In addition, this intervention is being targeted to treatment initiators to leverage the *fresh start* effect [42], a period of heightened motivation and attention. ART adherence instructions are initially salient for treatment initiators; however, over time, they fade from attention. Among adults with HIV in sub-Saharan Africa, forgetfulness is the most frequently reported barrier to maintaining high long-term adherence [43,44]. Therefore, treatment initiators may need subsequent support for adhering to the ART medication protocol until the behavior has been successfully routinized, which will be provided in the form of SMS text messages and incentives.

### Objective

We propose to test the intervention, *Incentives and Reminders to Improve Long-term Medication Adherence* (INMIND), in a pilot, parallel group randomized controlled trial (RCT) at the Mildmay Uganda HIV clinic in Kampala, Uganda, with 2 intervention groups and 1 control group, using an even allocation ratio of 1:1:1 among the 3 study groups. All (150/150, 100%) participants (including those in the control group) will receive information about the importance of behavioral routines, as it is a part of the standard adherence counseling for treatment initiators at Mildmay Uganda, and will create personalized ART adherence anchoring strategies. The first intervention group will additionally receive SMS text message reminders of their anchoring strategy, and the second intervention group will receive both SMS text message reminders and small incentives conditional on taking ART pills within a time window that corresponds to participants’ personalized anchoring strategy. We hypothesize that the SMS text message reminders and incentives will increase participants’ use of their anchoring strategy, and thus, medication adherence will be better maintained after the intervention ends in both the intervention groups relative to the control group. We also expect to see stronger maintenance in the intervention group receiving both reminder messages and incentives than that in the intervention group receiving only reminder messages.

## Methods

### Ethics Approval

This pilot RCT has been funded by the National Institute of Mental Health in the United States (R34MH122331) and approved by the RAND Corporation’s Human Subjects Protection Committee (2020-N0632), Mildmay Uganda Research Ethics Committee (0701-2021), and Uganda National Council for Science and Technology (HS128ES).

### Study Design

**Figure 2:**
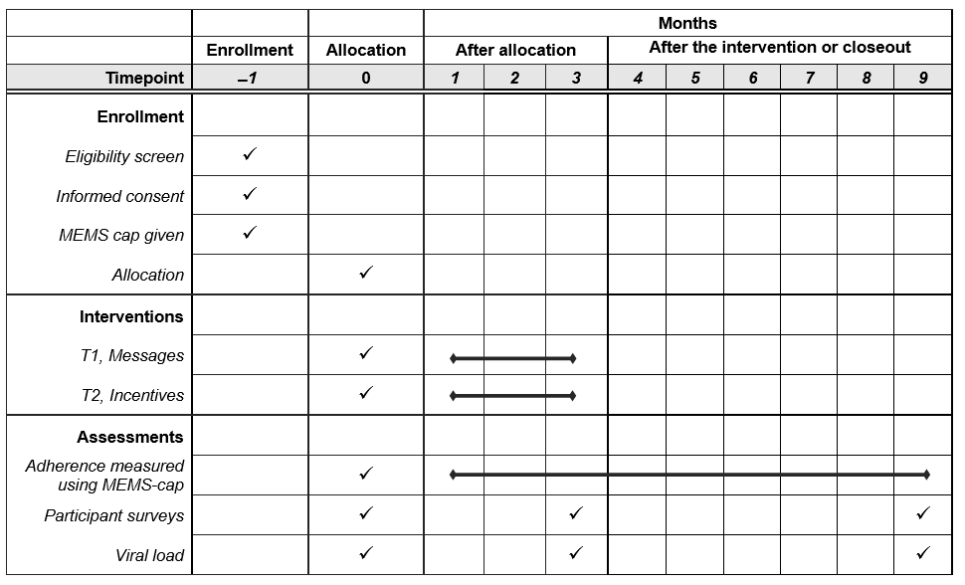
Schedule of enrollment, interventions, and assessments (SPIRIT [Standard Protocol Items: Recommendations for Interventional Trials] figure). MEMS: medication event monitoring system; T1: treatment group 1; T2: treatment group 2.

This study will use a 3-armed RCT (2 intervention groups and 1 control group) with randomization at the individual level. Refer to the SPIRIT (Standard Protocol Items: Recommendations for Interventional Trials) checklist for a guide to the key items reported in this protocol (Figure 2). The interventions will be administered for a period of 3 months from the baseline survey. During the intervention period, medication event monitoring system (MEMS) data readings will be collected monthly, wherein prize drawings for eligible participants in the *incentives* group will be conducted. As the population of interest is treatment initiators, the monthly study visits are expected to coincide with the monthly clinic visits as mandated for newly diagnosed clients who are becoming accustomed to ART treatment. During the postintervention period, the team will follow participants and continue MEMS data collection during the participants’ regular clinic visits (expected to be every month for the first 6 months after treatment initiation and every 1-3 months thereafter) for a period of 6 months, thus bringing the total study period to 9 months.

As the long-term goal of the study is viral suppression, viral load data will also be collected during the routine assessment. Viral load tests for treatment initiators are conducted after the first 6 months of ART initiation and then changed to 12-month periods as per the clinic and Ugandan Ministry of Health guidelines. Survey assessments will be administered at baseline, 3 months, and 9 months for all (150/150, 100%) participants.

It is important to note that the pilot study is being preceded by a formative phase and succeeded by an adaptation phase, both of which are designed to collect qualitative data pertaining to the overall design, feasibility, and acceptability of the interventions. The results of the formative phase will be used to adapt the pilot intervention materials and timeline before intervention administration. The adaptation phase is expected to provide feedback on several critical parameters of the interventions, which will be used to mold the intervention in preparation for a full-scale RCT. Figure 2 shows the timing of the study activities.

### Study Sites

The study will be performed at Mildmay, Uganda, an HIV clinic in Kampala, Uganda. This clinic specializes in providing comprehensive HIV and AIDS prevention, care, and treatment services to >105,000 patients with HIV. It offers integrated health services to a diverse population base; of the 15,000 patients served at the main site in Lweeza, 11% are children younger than 18 years, 65% are women, and 100% of the patients are on ART. Mildmay also has well-established electronic medical records infrastructure, making it one of the growing number of facilities using electronic medical record systems in Uganda.

Apart from the patient-facing work, Mildmay also provides technical assistance to organizations and governments, hosting training and educational courses for >1500 professionals per year. Furthermore, the Mildmay Uganda laboratory is accredited by the South African National Accreditation System under International Organization for Standardization 15,189:2012 and specializes in virology and other tests. Mildmay also has numerous ongoing research projects involving international researchers. It has a standing community advisory board comprising church leaders, elected councilmen, health care providers (external to Mildmay), and advocates of clients with HIV.

### Sampling Strategy

The objective of this study is to establish preliminary efficacy, acceptability, and feasibility of the interventions. Consequently, 150 participants will be recruited from Mildmay’s main site for the pilot study. The sample will be representative of the patient population at Mildmay, with an approximately 70:30 ratio of female to male clients.

Electronic medical records and the hospital electronic database will be used to screen the client population for initial eligibility based on age and ART initiation period, as noted in the eligibility criteria in the following sections. For this step, the hospital staff (and not the study coordinator) will mine the electronic database for this information to create a master list of eligible clients. Given the focus on treatment initiators, this process will be conducted weekly to identify newly diagnosed clients who are eligible to participate in the study.

A daily list of expected clients will be generated from the master list for the study coordinators to identify those deemed eligible and who are due for a clinic visit. Once a client is identified, a prebaseline visit will be initiated, with the study coordinator approaching the client and inquiring about their interest in participating in an ongoing study. On confirming interest, clients will be taken to a separate study room to verify their eligibility and to obtain their consent to participate. Consented participants will be given a MEMS cap and instructed to store one of their HIV medications in a pill bottle with the MEMS cap attached. In addition, they will be given a study appointment approximately 1 month after this initial visit for adherence data retrieval and baseline survey administration. The first month of adherence data will be used as a baseline, and the intervention will not begin before this follow-up visit.

### Inclusion Criteria

The study sample will consist of male and female clients aged ≥18 years who have started ART at Mildmay within the preceding 3 months. Treatment initiation is an important eligibility criterion given the conceptual framework of the pilot study, which suggests that habit formation during initiation is a key driver for sustained ART adherence over time. On the basis of electronic records data from Mildmay, each month, at least 100 to 150 clients with these characteristics begin treatment at the hospital (a total of 1758 clients in 2018); therefore, a large pool of potentially eligible clients will be available for recruitment. Given the nature of the intervention, inclusion will also require participants to own or have access to a phone for at least 5 days per week throughout the duration of the intervention and be willing to receive SMS text messages throughout the intervention period.

### Exclusion Criteria

Children aged <18 years were excluded for 2 reasons: (1) the pediatric clinic is separate from the main recruitment site (which primarily caters to adult clients) and (2) the intervention may require alteration to account for the specific needs of children and adolescents. In addition, clients who are not mentally fit to understand the consenting or study procedures and clients who speak neither English nor Luganda (the local language spoken by most people in and around Kampala) were excluded. As both intervention groups rely on receiving SMS text messages, clients who do not own a mobile phone or have access to one will also be excluded.

Participation in another adherence study and inability or nondesire to use MEMS caps regularly throughout the course of the study will also be a basis for exclusion. Consequently, if the baseline MEMS reading suggests that participants opened their pill bottle <30% of the days and if that was not a consequence of low adherence, the participant will be given a transport refund and be unenrolled from the study. Finally, clients who come outside regular clinic working hours will be excluded.

### Randomization and Allocation

Participants will be randomly assigned to either 1 of the treatment arms (treatment group 1 [T1]=*messages*; treatment group 2 [T2]=*incentives*) or the control arm after consenting but will only be informed of their assignment after the baseline survey is over to minimize any potential influence of the assignment on the baseline survey responses. The distribution ratios for the randomization will be 1:1:1, and the assignment will be conducted through a computer-generated randomization component built into the baseline survey administration software called Questionnaire Development System. The random assignment (to either the control arm or 1 of the 2 intervention arms) will be revealed at the end of the baseline survey to both the participant and study coordinator, who will not know the respondent’s treatment assignment during the survey. Given the nature of the intervention, neither the interviewers nor the participants can be blinded to the treatment status. However, the data analyst who will conduct the impact analysis will be blinded to treatment assignment.

### Procedures

#### Interventions

The pilot study will include a control arm, along with 2 intervention arms—*messages* (T1) and *incentives* (T2).

##### Control Group: Usual Care

Participants assigned to this arm will receive care as usual, including the adherence support mechanisms that are part of usual care practices. At recruitment, participants will be educated about the importance of pill taking using a leaflet that provides information on how to establish healthy pill-taking routines. Then, participants will be asked to select 1 of the following 3 pill-taking anchors: getting dressed in the morning, eating a meal (breakfast) in the morning, or eating dinner in the evening, which will also be described in a habit leaflet, and they will be asked to specify the time at which their anchor typically occurs. Once selected, participants will be asked to continue using their MEMS caps and bring them during the next visit.

During each of the subsequent study visits, scheduled approximately 1 month apart during the intervention period and 1 to 3 months apart subsequently, participants will be asked to complete a short questionnaire inquiring about changes in their ART adherence behavior, including any changes in location, times, and social activities surrounding their pill-taking behavior and ART adherence habit strength. They will also be asked about any clinical changes in their ART regimen and about their daily MEMS cap use. This is because pill pocketing (dispensing >1 dose in a given bottle opening event) is a common phenomenon among the population, with 15% of a previous study sample reporting pocketing [45]. If participants indicate pocketing, the study coordinator will work with the participant to find another solution to avoid pocketing and continue using their MEMS cap, and we will adjust for their pocketing in our assessment of their adherence outcomes. Finally, participants will be asked about any changes in their contact information or addresses, and updates will be noted in the contacts data.

Then, the study coordinator will download the readings from the MEMS cap, inquire about the participants’ next visit, and remind them to continue taking their pills on time. This procedure will be conducted throughout the intervention and postintervention periods.

##### T1—Messages Group

Participants assigned to this arm will receive daily SMS text messages in addition to care as usual during the 3-month intervention period. When the participant is informed about their treatment assignment, they will be educated using the same habit leaflet as all other study groups and asked to pick an anchor and share the time of the day the anchor typically occurs. In addition, the study coordinator will register the participant’s mobile phone number along with their language preference for SMS text messages (English or Luganda) on a web-based SMS text messaging system, Twilio. The system will include programmed SMS text messages that will be sent to all (50/50, 100%) participants at 2 PM local time every day for the 3-month intervention period. Textbox 1 shows some example SMS text messages, each of which would be sent on a specific day of the week. Once registered, the study coordinator will send the participant a test message to confirm registration. If, for any reason, the participant is unable to confirm receipt of the test message (eg, if their primary number is owned by another person whom they live with), the study coordinator will try registration again. If the registration is still not confirmed, the participant will be unenrolled from the study.

During each subsequent intervention-period study visit for the enrolled participants, they will be asked about the SMS text messages in addition to the questions asked to the participants in the control group. Specifically, participants will be asked whether they have been receiving the SMS text messages and whether they read them. MEMS data will also be downloaded during each subsequent clinic visit.

**Example SMS text messages for the messages and incentives groups in the Incentives and Reminders to Improve Long-term Medication Adherence (INMIND) intervention.**

**Sample messages**

- Hello, this is INMIND. Take your vitamins together with your existing routine for good health!
- Hello, this is INMIND. Forming routines requires effort now but will pay off in the end!
- Hello, this is INMIND. Don’t forget to take your vitamins every day at the same time!
- Hello, this is INMIND. Remember to stick with your healthy plans!

##### T2—Incentives Group

Participants assigned to this arm will receive monetary incentives in addition to daily SMS text messages (similar to the *messages* arm) and care as usual during the 3-month intervention period. Upon revealing their group assignment, participants will be registered on the Twilio platform following the same procedures outlined for the *messages* arm. Participants will also be informed that if, at their next visit, they have taken their medication on ≥70% of the past 30 days within –1 to +1 hour of their chosen anchor time, they will be eligible to participate in a lottery to win mobile airtime. The study coordinator will explain that they will receive 3 opportunities to participate in the lottery (ie, at each study visit over the next 3 months, if they are eligible). To ensure that participants understand the process, the study coordinator will conduct a mock prize drawing, wherein participants will choose 1 of 3 cards listing an airtime prize amount. The chosen card and corresponding prize will be revealed to the participant, and the amount listed on the card will be sent to the participant’s phone number (via Reloadly) in the form of a mobile top-up balance.

During each subsequent study visit, the study coordinator will check the client’s MEMS cap data and fill a form that asks questions about adherence behaviors (similar to the control group) and SMS text messages (similar to the messages group). The study coordinator will also ask about pill pocketing to assess prize eligibility. To avoid unfairly punishing participants who pocket doses (resulting in an underreporting of MEMS data in the software), participants will be asked about an estimated number of doses pocketed and will be allowed to enter a wild card prize drawing when they did not reach the adherence threshold. They will be specifically informed that this will be a 1-time exception. After the 3-month intervention, participants will follow the same procedures as the control and messages groups.

#### MEMS Cap Procedures

The MEMS caps will be used to assess the primary outcome measures—mean monthly adherence during the intervention and postintervention periods and a novel adherence measure assessing the timeliness of adherence during the intervention and postintervention periods. The caps will be distributed to all (50/50, 100%) participants, regardless of their group assignment to avoid any spurious intervention effects associated with cap use. The data captured by these caps will be downloaded at each clinic visit using a MEMS cap reader that will be connected to a study computer, and we will use these data to construct our participant-day-level measures of ART medication adherence.

The study coordinator will assist the patient in dispensing their medication into a bottle that we provide with an attached MEMS cap, or, if preferred by the patient, they can put the MEMS cap on the medication bottle provided by the clinic. We will monitor adherence to only 1 daily dose of antiretroviral medication, as studies show that rates of adherence do not differ significantly across medications taken by an individual patient [46]. All (150/150, 100%) participants will be asked to use their MEMS cap continuously throughout the study and return with the cap and pill bottle for each clinic visit. Participants will be informed that the cap records when the bottle is opened. They will also be informed that these data will not be shared with clinicians.

#### Study Timeline

##### Recruitment

The recruitment visit (or prebaseline visit) will be used as an opportunity to screen the clients. Once the client is found to meet the initial eligibility criteria, they will be given a MEMS cap, which will be used to track baseline adherence and study eligibility for approximately 1 month. If their clinic visit does not coincide with the expected study visit, participants will be provided a study visit appointment and will be told that they will receive travel compensation for making the additional trip. Clients will also be asked to consent to participate during this initial recruitment visit.

##### Visits Between Month 1 and Month 3

During the 3-month intervention period, participants will be scheduled for monthly clinic appointments, and the study coordinators will (1) collect information on changes or degradations of existing routine behaviors, (2) conduct prize drawings with the *incentive* group, and (3) update contact information if phone numbers or addresses have changed. After the first 3 months, this information will be collected during the participants’ regular clinic visits. When participants report degradation of their selected behavioral anchor, a new daily routine will be identified for the anchoring strategy from the suggested list of 3 common routine behaviors.

##### Postintervention Surveys

Follow-up surveys will be administered at month 3 (ie, at the end of the intervention) and month 9 (ie, 6 months after the end of the intervention) to evaluate behavioral persistence. These surveys will be designed to evaluate how the intervention affects both primary and secondary outcomes and cognitive and motivational factors that may be influenced by the intervention. Intervention effects over the 9-month study period will also be assessed, as these effects may be most pronounced in the first months when the pill-taking routine is first established but taper as the novelty of the intervention fades or anchoring routines are changed. The postintervention assessment will happen during a scheduled hospital visit to avoid participants having to make costly additional hospital visits specifically for interviews.

#### Data Collection Methods

##### Surveys

The baseline survey contains questions pertaining to the following:

1. *Demographics and socioeconomic status*, including age, sex, education, relationship status, employment type and status, income, preferred language, housing, economic shocks, food insecurity, and household composition
2. *Attitudes and beliefs about HIV medication*, including adherence behaviors, perceived benefits, and community perceptions around pill taking
3. *Sources of motivation* for medication adherence; for example, reduction in HIV transmission, maintenance of good health and ability to look after family, or maintenance of good physical appearance
4. *Habits* associated with regular pill taking, including pill-taking routines, missed doses and reasoning (to assess any perceptual and structural barriers to pill taking), and costs associated with prescription fills. This will be assessed using the Self-Reported Behavioral Automaticity Index [47], which is a validated subset of the Self-Reported Habit Index [48] that measures the automaticity of performing a specific routine behavior based on responses to 4 questions (eg, “taking my medication is something I do without thinking”) on a 7-point Likert scale ranging from “strongly disagree” to “strongly agree.”
5. *Health care information*, including perceptions about health since initiating ART and over the past month

In addition, a measure developed by Falk et al [49] will be used to assess risk and time preferences, altruism, trust, and positive and negative reciprocity on a scale from 0 to 10, where 0 means “completely unwilling to take risks” and 10 means “very willing to take risks.” In addition, the Intrinsic Motivation Inventory will be used to examine participants’ subjective motivation for taking medications [50]. The 3-month and 9-month surveys will additionally include questions assessing comfort with using the MEMS caps, acceptability of the SMS text messages and prize drawings, and overall study satisfaction. Upon completing each survey, participants will be given USh 30,000 (approximately US $8.25) as travel compensation.

##### MEMS Data and Access Forms

The MEMS data will be automatically downloaded and stored electronically using the MEMS cap software that will be installed on a tablet accessible by the study coordinator. Other study data, including participants’ survey responses, monthly visit reports, and other information about study dropout, will be recorded by study personnel in Microsoft Access templates designed by the research team. These electronic data will be safely stored at Mildmay and securely transferred to the research team in the United States periodically during the study period and at the end of the 9-month study.

#### Chart Abstraction

Participant’s viral load will be used as a complementary measure to the MEMS data for the assessment of ART adherence. They are now a part of routine clinical care at Mildmay, with tests conducted when someone receives a positive HIV test result, after 6 and 12 months from the initial diagnosis, and every 12 months thereafter. Consequently, the results of the viral load test will be collected from electronic medical records, and the data abstraction will be timed with the routine tests for the participants.

#### Participant Tracking

Extensive tracking information will be collected at recruitment and will be verified at each study visit. This will include the participants’ mobile phone numbers, home addresses, and email addresses and contact information of 2 individuals who live in the same community and whom the participant is comfortable and familiar with. These additional contacts are collected to ensure that the participant can be located if their contact information changes. These procedures have limited attrition in the researchers’ previous studies to approximately 5% [45,51].

If a participant in either treatment group loses, breaks, or cannot use their mobile phone, they will no longer be able to receive SMS text messages as part of the intervention. To address this possibility, the study coordinator will record the functionality of participants’ mobile phones at each monthly visit, and those with missing or broken mobile phones will be noted. In addition, all active phone numbers of the participants (as it is not unusual for Ugandan people to use >1 SIM card or phone) will be recorded to ensure that an alternative means of delivering the messages exists, when available. In addition, participants will be given a handout at the start of the study, explaining that they should give the study team a call if they change phones or phone numbers at any point during the first 3 months of the study and that they will be rewarded with USh 3000 (US $0.78) for notifying the study coordinators of such a change.

### Outcomes

The study will assess the following outcomes.

#### Feasibility and Acceptability

The feasibility and acceptability of the INMIND intervention will be assessed using the study retention rate, attendance rate for scheduled clinic visits, and survey responses collected at the end of the 3-month intervention and following the 6-month postintervention period. These survey measures ask participants about their ability to understand all the intervention materials and their perceived value of the intervention. We will also conduct focus groups with a sample of study participants after the postintervention survey to collect additional information about the study’s feasibility and acceptability.

#### Preliminary Efficacy

##### Primary Outcome 1: Electronically Measured Mean Medication Adherence During the Intervention

MEMS data will be collected continuously over the course of the 3-month intervention period, allowing for mean adherence assessment during the intervention period. Specifically, the number of pill bottle openings detected by the MEMS cap will be used as a measure of each participant’s pills taken per day. Then, mean adherence will be calculated as the number of pills taken per day during the intervention period divided by the number of pills prescribed during the intervention for a given participant (ie, number of actual bottle openings/number of prescribed bottle openings). This mean adherence measure will be capped at 100%, meaning that any pill bottle openings beyond the participants’ number of prescribed daily pills will be ignored. In this way, the mean adherence measure will range from 0 to 100 and will be calculated for each participant on each day of the intervention period. Only one of the daily ART medication doses will be used to calculate the primary mean adherence variable. Both mean adherence over the 3-month intervention and monthly measure of mean adherence will be calculated and analyzed.

##### Primary Outcome 2: Electronically Measured Mean Medication Adherence After the Intervention

After the intervention, MEMS data will be collected continuously for 6 months to investigate postintervention mean ART adherence (*persistence*). The calculation of this outcome is the same as that of *outcome 1*, except for the timeline over which the data will be collected and analyzed.

##### Primary Outcome 3: Routinization of ART Adherence During the Intervention

In addition to *outcomes 1 and 2*, a novel measure of routine adherence will be assessed during the intervention period. The novelty of the measure is that it is explicitly based on the temporal pattern of pill taking. The measure will be calculated as the fraction of scheduled pills taken within a 2-hour window (–1 to +1 hour) around the typical time that participants report completing their existing routine behavior that anchors their pill taking. This measure provides an objective way for determining the behavioral automaticity of pill taking and will be calculated as the mean of all prescribed pills taken around the participants’ anchor time over the 3-month intervention.

##### Primary Outcome 4: Routinization of ART Adherence After the Intervention

The fourth outcome will be calculated using the same procedures as that for *outcome 3*. However, the data from which the measure will be assessed will be collected during the postintervention period.

The team will also assess 2 secondary efficacy measures, as described in the following sections.

##### Secondary Outcome 1: Retention in Care

Retention in care is an important metric of treatment adherence. Failure to remain in care is a commonly observed problem for treatment initiators. To assess how well participants will continue to remain in care, Mildmay electronic records will be used to evaluate the number of study participants who fail to attend their regularly scheduled clinic visits. Participants who do not make any clinic visits for ≥6 months will be considered lost to follow-up. The study coordinator will call them using the tracking information collected for the study to inquire about their reason for stopping care at the Mildmay clinic. This outcome will be measured as an indicator of whether the participants are still active clients at the clinic at the end of the 9-month study.

##### Secondary Outcome 2: Viral Suppression

HIV RNA (viral load) is our final outcome measure. According to the AIDS Clinical Trials Group, virological failure is defined as a confirmed viral load >200 copies/mL. Below this level, the viral load is considered undetectable. Importantly, this is a reliable biological measure of ART adherence, as strong adherence leads to low viral load. Given the intervention design, viral load will be an important complementary measure of adherence. The analysis will examine the intervention’s effects on the likelihood of being virally suppressed at the end of the 9-month study period.

### Data Analysis

#### Statistical Methods and Analyses

Feasibility and acceptability will be analyzed using summary statistics derived directly from our self-reported measures. To estimate preliminary efficacy, statistical analyses comparing group-level differences in the secondary and tertiary outcome measures will be performed. An analysis of covariance framework will be used to test for group differences in each secondary and tertiary outcome, controlling for the participant characteristics that are found to differentiate the groups at baseline. For analyses of dichotomous variables, such as viral suppression, nonparametric McNemar test and analogous multiple logistic regression will be used to control for covariates to assess group differences. In addition to static comparisons of group means for each outcome at 3-month intervals, the longitudinal nature of the data will be leveraged by using repeated measures and time series techniques. Specifically, a linear mixed model with repeated observations will be fit using maximum likelihood through *xtmixed* in the software package, Stata, to study group-level temporal dynamics in daily measures of the primary and secondary outcomes.

#### Sample and Effect Size Estimation

We will recruit a total of 150 participants and will assume a 90% retention rate throughout the 9-month study period. This is a conservative estimate of attrition, as previous studies in the same clinic and with a similar population observed only 6% attrition over 12-month study periods [45,51]. Given the study objectives of establishing acceptability, feasibility, and preliminary effectiveness, the targeted sample size may not have the power to detect many of the effects that would be clinically significant. Nonetheless, effect size calculations associated with 80% power (2-tailed *t* test) regarding the primary outcomes at month 9 have been performed. A recent study conducted at Mildmay suggested that mean ART adherence rates are approximately 75% (SD 20%), as measured by MEMS caps [51]. Using these parameters as estimates of adherence for the control group and 10% attrition at month 9, a sample size of 150 will provide sufficient power to detect 9% difference in mean adherence between the pooled intervention groups and the control group (effect size=0.47) and approximately 11% difference in mean adherence between the 2 intervention groups. This translates to the study being able to detect medium effect sizes.

#### Qualitative Analysis

Qualitative analysis will be primarily performed through semistructured interviews and focus groups with clients, providers, and clinic administrators during the initiation and adaptation phases. The interviews administered during the initiation phase will primarily focus on perceptions of ART pill taking as an activity of daily life and investigate the range of existing behaviors that can be used as cues (such as eating, daily prayers, or brushing teeth) to understand how INMIND can best support ART adherence routinization. During the adaptation phase, 8 focus groups (n=4, 50% among participants in the *messages* group and n=4, 50% among the participants in the *incentives* group) will be organized to elicit additional qualitative information about areas of program improvements that may not be captured in the surveys.

All qualitative data will be digitally recorded; translated into English; and uploaded to Dedoose, an analytic software package. In total, 2 qualitative researchers will independently read the text and identify all topics covered. A codebook that describes the inclusion and exclusion criteria, along with typical exemplars for each topic or theme will be developed. Intercoder reliability (evidenced by Cronbach α≥.70) will be established; discussion will be conducted until the coders converge on a single, agreed-upon meaning for the thematic area. For each topic, they will identify the range of themes, with attention to the most commonly discussed (ie, key themes) and least frequently discussed (ie, outliers) themes. They will produce research reports on specific topics describing the range, central tendency, and distribution of each theme.

### Data Management

All data collected during the course of the study will have only the existing clinic identifiers as the unique participant identifiers. All other identifiable information will be stored separately. Data collection and storage hardware (ie, tablets and computers) will be password-protected, and physical storage spaces will have a locking mechanism for security. All physical storage spaces will be located at the Mildmay RAND office in Kampala, with access granted only to key personnel and the principal investigator. These physical storage spaces will be used to store the consent forms and other physical tracking documentation.

All data collection and on-ground study activities will be conducted by the study team in Uganda. This team will include a team leader, 2 lead interviewers, and 3 supporting team members. The design of the data collection instruments and protocols, quality monitoring of the qualitative and quantitative data, and data analysis will be conducted by the study team based in the United States. Data collected on the ground will be transferred to the US team on a weekly basis through a secure web portal (Kiteworks).

Published material will not contain any personally identifying information. There is no data monitoring committee because the trial was deemed to have minimal risk. The study team in the United States will still perform data monitoring and quality assurance exercises weekly during the 9-month study period.

### Handling Missing Data and Attrition

Missing data for some variables will be imputed if a participant remained enrolled in the study. When participants drop out, multiple logistic regression models will be fit to assess whether this dropout is random. If it is not, *nonresponse* weights using logistic regression will be developed to adjust for dropout. All analyses will reflect these design effects in the calculation of SEs and statistical tests of significance*.*

## Results

Recruitment was completed as of February 18, 2022. A total of 150 participants were recruited, and data collection is expected to end in December of 2022. Final results are expected to be submitted for publication by April 2023.

## Discussion

### Expected Outcomes

We hypothesize that our 2 intervention groups will display high mean medication adherence and high routinized medication adherence (ie, pill taking within –1 to +1 hour of participants’ chosen anchor) during the 3-month intervention relative to the control group. After using their anchoring strategy more frequently, we hypothesize that our 2 intervention groups will better maintain their mean medication adherence and routinized medication adherence over the 6 months after the intervention is withdrawn. Finally, we hypothesize that our second treatment group, which receives both SMS text message reminders and small incentives for using their anchor during the 3-month intervention, will more strongly maintain their mean medication adherence and routinized medication adherence during the 6-month postintervention period.

### Comparison With Previous Studies

INMIND has a strong scientific premise that addresses a critical knowledge gap in the literature around the design of interventions for establishing and maintaining long-term behavior change. A growing body of literature in the field of psychology targets long-term behavior change [18,52]; however, most intervention methods are one-off interventions that do not support participants during the approximately 3-month routinization process [17,20,53,54]. Behavioral economics–based interventions have also had limited efficacy in maintaining long-term behavior change. For example, incentives have successfully changed a range of health behaviors by countering present bias [38-41], including improved ART adherence [55], but these interventions typically show only short-term impact that dissipates after the incentives are withdrawn [38,52]. In our own previous studies, we also found that incentives did not have persistent effects, and only participants who showed timely adherence (an indicator that they potentially anchored pill taking to an existing routine) maintained high adherence after the incentives were withdrawn [51].

Our combined intervention approach attempts to leverage the successful components of these existing psychology-based and behavioral economics–based interventions to better maintain ART adherence. If successful, this intervention will help to expand the understanding of the habit formation process and common psychological barriers to successfully adhering to new health behaviors. The study also addresses biological variables such as age and sex appropriately and incorporates them in both the impact analysis and when testing for age and sex differences in behavioral biases and intrinsic motivation. Such analyses will be especially useful in designing a large-scale intervention that can assist all treatment initiators in establishing and maintaining long-term ART adherence.

### Limitations

The study has some limitations. First, the study will recruit the sample from 1 clinic in Uganda, limiting the generalizability of our results. Second, the small sample size limits the team’s ability to detect clinically meaningful effect sizes. Although this is not the intended aim of this feasibility and acceptability study, the small sample size is still a limitation to the statistical analyses. Third, ART adherence will only be measured over 9 months; therefore, future studies will be needed to assess ART adherence over long durations. Fourth, ART adherence will be measured using MEMS caps, which is currently one of the most accurate ways to measure medication adherence, but conscious manipulation of the pill bottle openings by the participants is still a possibility that may lead to an overestimation of the study’s adherence outcomes for participants in the *incentives* group, who may use deception to increase their chances of a prize drawing.

### Dissemination and Future Directions

The team will use peer-reviewed publications and conference presentations as the primary means of disseminating results. The findings will be relevant to those interested in the behavioral mechanisms that underlie successful long-term ART adherence and, more broadly, the mechanisms underlying long-term behavior change. In addition, the findings will be used in the design of a large-scale RCT that we will use to rigorously assess the effectiveness of the INMIND intervention for establishing long-term ART adherence habits. In addition to future RCTs, we plan to use detailed MEMS data on the timing of daily pill taking to inform statistical models of the habit formation process, which will guide new intervention designs for promoting ART adherence habits.

## Appendix

Multimedia Appendix 1 Peer-review report from the HIV/AIDS Intra- and Inter-personal Determinants and Behavioral Interventions Study Section, AIDS and Related Research Integrated Review Group, Center for Scientific Review (National Institutes of Health, USA).

## Abbreviations

ART: antiretroviral therapy
INMIND: Incentives and Reminders to Improve Long-term Medication Adherence
MEMS: medication event monitoring system
RCT: randomized controlled trial
SPIRIT: Standard Protocol Items: Recommendations for Interventional Trials
T1: treatment group 1
T2: treatment group 2

## Data Availability

Deidentified research data will be made available, along with the survey instruments, to interested external researchers through collaborative agreements with the principal investigator and coinvestigators, as required by the National Institutes of Health’s data sharing policies.

## References

[1] Palella Jr FJ, Delaney KM, Moorman AC, Loveless MO, Fuhrer J, Satten GA, Aschman DJ, Holmberg SD (1998). Declining morbidity and mortality among patients with advanced human immunodeficiency virus infection. HIV Outpatient Study Investigators. N Engl J Med.

[2] Parienti JJ, Ragland K, Lucht F, de la Blanchardière A, Dargère S, Yazdanpanah Y, Dutheil JJ, Perré P, Verdon R, Bangsberg DR, ESPOIR and REACH study groups (2010). Average adherence to boosted protease inhibitor therapy, rather than the pattern of missed doses, as a predictor of HIV RNA replication. Clin Infect Dis.

[3] Kobin AB, Sheth NU (2011). Levels of adherence required for virologic suppression among newer antiretroviral medications. Ann Pharmacother.

[4] Pasternak AO, de Bruin M, Jurriaans S, Bakker M, Berkhout B, Prins JM, Lukashov VV (2012). Modest nonadherence to antiretroviral therapy promotes residual HIV-1 replication in the absence of virological rebound in plasma. J Infect Dis.

[5] (2017). AIDSinfo. Joint United Nations Programme on HIV/AIDS.

[6] Nachega JB, Marconi VC, van Zyl GU, Gardner EM, Preiser W, Hong SY, Mills EJ, Gross R (2011). HIV treatment adherence, drug resistance, virologic failure: evolving concepts. Infect Disord Drug Targets.

[7] May MT, Gompels M, Delpech V, Porter K, Orkin C, Kegg S, Hay P, Johnson M, Palfreeman A, Gilson R, Chadwick D, Martin F, Hill T, Walsh J, Post F, Fisher M, Ainsworth J, Jose S, Leen C, Nelson M, Anderson J, Sabin C, UK Collaborative HIV Cohort (UK CHIC) Study (2014). Impact on life expectancy of HIV-1 positive individuals of CD4+ cell count and viral load response to antiretroviral therapy. AIDS.

[8] Hardon AP, Akurut D, Comoro C, Ekezie C, Irunde HF, Gerrits T, Kglatwane J, Kinsman J, Kwasa R, Maridadi J, Moroka TM, Moyo S, Nakiyemba A, Nsimba S, Ogenyi R, Oyabba T, Temu F, Laing R (2007). Hunger, waiting time and transport costs: time to confront challenges to ART adherence in Africa. AIDS Care.

[9] Tuller DM, Bangsberg DR, Senkungu J, Ware NC, Emenyonu N, Weiser SD (2010). Transportation costs impede sustained adherence and access to HAART in a clinic population in southwestern Uganda: a qualitative study. AIDS Behav.

[10] Byakika-Tusiime J, Oyugi JH, Tumwikirize WA, Katabira ET, Mugyenyi PN, Bangsberg DR (2005). Adherence to HIV antiretroviral therapy in HIV+ Ugandan patients purchasing therapy. Int J STD AIDS.

[11] Gallant JE (2000). Strategies for long-term success in the treatment of HIV infection. JAMA.

[12] Shuter J, Sarlo JA, Stubbs RO, Rode RA, Zingman BS (2012). Sequential antiretroviral adherence measurement using electronic bottle cap monitors in a cohort of HIV-infected adults. J Int Assoc Physicians AIDS Care (Chic).

[13] Ortego C, Huedo-Medina TB, Llorca J, Sevilla L, Santos P, Rodríguez E, Warren MR, Vejo J (2011). Adherence to highly active antiretroviral therapy (HAART): a meta-analysis. AIDS Behav.

[14] Gardner EM, McLees MP, Steiner JF, Del Rio C, Burman WJ (2011). The spectrum of engagement in HIV care and its relevance to test-and-treat strategies for prevention of HIV infection. Clin Infect Dis.

[15] Alison Phillips L, Leventhal H, Leventhal EA (2013). Assessing theoretical predictors of long-term medication adherence: patients' treatment-related beliefs, experiential feedback and habit development. Psychol Health.

[16] Brooks TL, Leventhal H, Wolf MS, O'Conor R, Morillo J, Martynenko M, Wisnivesky JP, Federman AD (2014). Strategies used by older adults with asthma for adherence to inhaled corticosteroids. J Gen Intern Med.

[17] Wood W, Neal DT (2007). A new look at habits and the habit-goal interface. Psychol Rev.

[18] Lally P, Gardner B (2013). Promoting habit formation. Health Psychol Rev.

[19] Rothman AJ, Sheeran P, Wood W (2009). Reflective and automatic processes in the initiation and maintenance of dietary change. Ann Behav Med.

[20] Redish AD, Jensen S, Johnson A (2008). Addiction as vulnerabilities in the decision process. Behav Brain Sci.

[21] Duhigg C (2013). The Power of Habit: Why We Do What We Do, and How to Change.

[22] Pfeffer I, Strobach T (2018). Behavioural automaticity moderates and mediates the relationship of trait self-control and physical activity behaviour. Psychol Health.

[23] Gardner B (2015). A review and analysis of the use of 'habit' in understanding, predicting and influencing health-related behaviour. Health Psychol Rev.

[24] Rothman AJ, Gollwitzer PM, Grant AM, Neal DT, Sheeran P, Wood W (2015). Hale and hearty policies: how psychological science can create and maintain healthy habits. Perspect Psychol Sci.

[25] Park DC, Kidder DP, Brandimonte M, Einstein GO, McDaniel MA (1996). Prospective memory and medication adherence. Prospective Memory: Theory and Applications.

[26] Prestwich A, Lawton R, Conner M (2003). The use of implementation intentions and the decision balance sheet in promoting exercise behaviour. Psychol Health.

[27] Achtziger A, Gollwitzer PM, Sheeran P (2008). Implementation intentions and shielding goal striving from unwanted thoughts and feelings. Pers Soc Psychol Bull.

[28] Armitage CJ, Arden MA (2008). How useful are the stages of change for targeting interventions? Randomized test of a brief intervention to reduce smoking. Health Psychol.

[29] Sheeran P, Orbell S (1999). Implementation intentions and repeated behaviour: augmenting the predictive validity of the theory of planned behaviour. Eur J Soc Psychol.

[30] Lally P, Chipperfield A, Wardle J (2008). Healthy habits: efficacy of simple advice on weight control based on a habit-formation model. Int J Obes (Lond).

[31] Gardner B, de Bruijn GJ, Lally P (2011). A systematic review and meta-analysis of applications of the Self-Report Habit Index to nutrition and physical activity behaviours. Ann Behav Med.

[32] Verplanken B, Faes S (1999). Good intentions, bad habits, and effects of forming implementation intentions on healthy eating. Eur J Soc Psychol.

[33] Beeken RJ, Croker H, Morris S, Leurent B, Omar R, Nazareth I, Wardle J (2012). Study protocol for the 10 Top Tips (10TT) trial: randomised controlled trial of habit-based advice for weight control in general practice. BMC Public Health.

[34] Enriquez M, McKinsey DS (2011). Strategies to improve HIV treatment adherence in developed countries: clinical management at the individual level. HIV AIDS (Auckl).

[35] Gardner B, Sheals K, Wardle J, McGowan L (2014). Putting habit into practice, and practice into habit: a process evaluation and exploration of the acceptability of a habit-based dietary behaviour change intervention. Int J Behav Nutr Phys Act.

[36] O'Donoghue T, Rabin M (2015). Present bias: lessons learned and to be learned. Am Econ Rev.

[37] Chetty R, Looney A, Kroft K (2009). Salience and taxation: theory and evidence. Am Econ Rev.

[38] Mantzari E, Vogt F, Shemilt I, Wei Y, Higgins JP, Marteau TM (2015). Personal financial incentives for changing habitual health-related behaviors: a systematic review and meta-analysis. Prev Med.

[39] Volpp KG, John LK, Troxel AB, Norton L, Fassbender J, Loewenstein G (2008). Financial incentive-based approaches for weight loss: a randomized trial. JAMA.

[40] Volpp KG, Loewenstein G, Troxel AB, Doshi J, Price M, Laskin M, Kimmel SE (2008). A test of financial incentives to improve warfarin adherence. BMC Health Serv Res.

[41] Charness G, Gneezy U (2009). Incentives to exercise. Econometrica.

[42] Dai H, Milkman KL, Riis J (2014). The fresh start effect: temporal landmarks motivate aspirational behavior. Manag Sci.

[43] Hodgson I, Plummer ML, Konopka SN, Colvin CJ, Jonas E, Albertini J, Amzel A, Fogg KP (2014). A systematic review of individual and contextual factors affecting ART initiation, adherence, and retention for HIV-infected pregnant and postpartum women. PLoS One.

[44] Buscher A, Hartman C, Kallen MA, Giordano TP (2012). Impact of antiretroviral dosing frequency and pill burden on adherence among newly diagnosed, antiretroviral-naive HIV patients. Int J STD AIDS.

[45] Linnemayr S, Stecher C, Mukasa B (2017). Behavioral economic incentives to improve adherence to antiretroviral medication. AIDS.

[46] Cramer J, Vachon L, Desforges C, Sussman NM (1995). Dose frequency and dose interval compliance with multiple antiepileptic medications during a controlled clinical trial. Epilepsia.

[47] Gardner B, Abraham C, Lally P, de Bruijn GJ (2012). Towards parsimony in habit measurement: testing the convergent and predictive validity of an automaticity subscale of the Self-Report Habit Index. Int J Behav Nutr Phys Act.

[48] Verplanken B, Orbell S (2003). Reflections on past behavior: a self-report index of habit strength. J Appl Soc Psychol.

[49] Falk A, Becker A, Dohmen T, Enke B, Huffman D, Sunde U (2018). Global evidence on economic preferences. Q J Econ.

[50] Deci EL, Ryan RM, Weiner IB, Craighead WE (2010). Intrinsic motivation. The Corsini Encyclopedia of Psychology.

[51] Stecher C, Mukasa B, Linnemayr S (2021). Uncovering a behavioral strategy for establishing new habits: evidence from incentives for medication adherence in Uganda. J Health Econ.

[52] Wood W, Neal DT (2016). Healthy through habit: interventions for initiating and maintaining health behavior change. Behav Sci Policy.

[53] Galla BM, Duckworth AL (2015). More than resisting temptation: beneficial habits mediate the relationship between self-control and positive life outcomes. J Pers Soc Psychol.

[54] Sheeran P (2002). Intention—behavior relations: a conceptual and empirical review. Eur Rev Soc Psychol.

[55] Linnemayr S, Hanoch Y, Barnes AJ, Rice T (2017). Behavioral economics and HIV: a review of existing studies and potential future research areas. Behavioral Economics and Healthy Behaviors: Key Concepts and Current Research.

