# Supplementary material for: Incentives and Reminders to Improve Long-term Medication Adherence (INMIND): Protocol for a Pilot Randomized Controlled Trial
Source: JMIR Res Protoc. 2022 Oct 31;11(10):e42216. doi: 10.2196/42216 (PMC9664328; doi:10.2196/42216)
Supplement: Multimedia Appendix 1 [file resprot_v11i10e42216_app1.pdf]

SUMMARY STATEMENT
(Privileged Communication)

PROGRAM CONTACT:
Michael Stirratt
240-627-3875
stirrattm@mail.nih.gov

Release Date: 03/31/2020
Revised Date:

Application Number: 1 R34 MH122331-01A1

Principal Investigator

LINNEMAYR, SEBASTIAN

Applicant Organization: RAND CORPORATION

Review Group: HIBI
HIV/AIDS Intra- and Inter-personal Determinants and Behavioral Interventions Study Section
AIDS

Meeting Date: 03/12/2020
Council: MAY 2020
Requested Start: 07/01/2020
RFA/PA: PA18-276
PCC: 9A-ASGA

Project Title: Incentives and ReMINDers to Improve Long-term Medication Adherence (INMIND)

SRG Action: Impact Score:35 Percentile:16 +
Next Steps: Visit https://grants.nih.gov/grants/next_steps.htm
Human Subjects: 30-Human subjects involved - Certified, no SRG concerns
Animal Subjects: 10-No live vertebrate animals involved for competing appl.
Gender: 1A-Both genders, scientifically acceptable
Minority: 5A-Only foreign subjects, scientifically acceptable
Age: 3A-No children included, scientifically acceptable

Project Year	Direct Costs Requested	Estimated Total Cost
1	175,000	269,078
2	150,000	230,638
3	125,000	192,198
TOTAL	450,000	691,914

ADMINISTRATIVE BUDGET NOTE: The budget shown is the requested budget and has not been adjusted to reflect any recommendations made by reviewers. If an award is planned, the costs will be calculated by Institute grants management staff based on the recommendations outlined below in the COMMITTEE BUDGET RECOMMENDATIONS section.

1R34MH122331-01A1 Linnemayr, Sebastian

RESUME AND SUMMARY OF DISCUSSION: This application proposes to develop the “INcentives and ReMINDers to Improve Long-term Medication Adherence (INMIND)” intervention using the ADAPT-ITT framework and then assess the feasibility and acceptability of the intervention. The INMIND intervention will use small incentives and daily text messages to help study participants recruited from a Ugandan HIV clinic to anchor their pill taking to an existing routine. A small randomized trial will be performed to test the two elements of the intervention compared to a control group. All study participants will receive standard information on establishing behavioral routines and adherence counseling. One intervention group will receive daily text messages reminding them to stick to their pill-taking anchor plan for three months. The other intervention group will receive the same text messages plus conditional awards for three months. The study outcomes will be medication adherence, retention in care and viral load after 12 months. In addition, qualitative data about the intervention will be gathered from stakeholders and participants to prepare for a future R01 application. Maintaining mediation adherence over time is challenging for most people and interventions that improve ART adherence have the potential to have a significant public health impact. The applicant has been responsive to concerns raised in the prior review of this application. The study team was strengthened by the addition of Dr. Yvonne Karamagi, a clinician. The intervention was refocused around behavioral anchors and incentives which the committee felt was a much stronger approach. The applicant also clarified a number of other areas that had raised questions in the prior review. However, the committee was not convinced of the need for incentives, although the combination with anchoring was seen as a potentially useful approach. There was also an issue raised about whether the study’s target population was young, male clients of the clinic. These remaining issues somewhat limited the committee’s overall enthusiasm for this much improved resubmission.

DESCRIPTION (provided by applicant): Recently the number of people initiating antiretroviral (ART) treatment (“treatment initiators”) has increased but too many fail to achieve viral suppression. Healthy routines are key to achieving long-term behavioral change and healthy outcomes, but few people manage to form them on their own. Existing interventions typically suggest that people anchor the targeted behavior to an existing routine, but fail to support participants during the time it takes to turn the behavior into a routine, with the result that typically fewer than half end up carrying the targeted behavior out automatically. Behavioral economics (BE) points to two important biases preventing many people from translating their good intentions into successful routines; it also suggests two readily implementable approaches to counter these same biases: the bias of lack of salience of chronic treatment adherence (i.e. over time, the more pressing needs of daily life dominate good intentions) can be countered by low-cost reminder messages sent via mobile phone during the time it takes to turn pill-taking into a routine behavior. Present bias (i.e. giving in to short-term temptations, which can lead to skipping pill doses) explains why many people have trouble sticking to their good intentions. Using small, intermittent incentives until the behavior becomes a routine is a novel approach that has the potential to be a game changer for establishing the (currently elusive) goal of long-term high ART adherence. The proposed R34 study will be implemented in a Ugandan HIV clinic in a 12-month randomized controlled trial (RCT) among treatment initiators to establish feasibility, acceptability, and preliminary efficacy of the intervention. Following formative work in Phase 1, all study participants will be told about the importance of routine pill-taking and receive a leaflet with strategies for anchoring pill-taking to an existing routine. Participants in the first intervention group (n=50) will then receive daily text messages for 3 months to reinforce that information (Message group). Participants in the second intervention group (n=50) will receive the same messages, but will also have a chance of winning small rewards conditional on high and timely medication adherence (Incentive group). Participants in the Control group will receive the usual standard of care. Persistence of adherence (primary outcome) and timely pill- taking (secondary outcome) will be measured using MEMS caps for 9 months after the 3-

months intervention, and retention in care as well as viral loads (secondary outcomes) will be assessed at month 12. Specific Aim 1 will be to evaluate the feasibility and acceptability of INMIND and develop the intervention using the ADAPT- ITT framework. Based on these insights, Specific Aims 2a and 2b will test the preliminary effectiveness of the intervention, including the relative effectiveness of two different implementation approaches (i.e. text messages alone vs. together with small BE-based incentives). Specific Aim 3 will collect data allowing adaptation of intervention parameters for a subsequent R01 application to test the intervention at scale.

PUBLIC HEALTH RELEVANCE: For public health it is important to support the growing number of ART treatment-initiating clients who often fail to achieve viral suppression, with catastrophic consequences in particular in resource-constrained countries in which treatment options are limited. This study aims to increase ART adherence among treatment initiators by anchoring pill-taking to an existing routine behavior with the help of small incentives based on principles from behavioral economics in combination with text messages sent by mobile phone. The approach is particularly targeted at those with low motivation and cognitive problems, thereby reducing health inequality and facilitating high, long-term ART adherence for a particularly vulnerable group of HIV clients.

CRITIQUE 1

Significance: 3
Investigator(s): 2
Innovation: 3
Approach: 3
Environment: 2

Overall Impact: This is a revised application to adapt and pilot an intervention to support ART adherence. The proposal aims to promote anchoring as a way to establish sustainable adherence routines. Grounded in psychological and behavioral economic theory, the investigators argue that using incentives to routinize adherence among initiators early on can lead to sustained adherence over time. The proposed study could generate more evidence for the role of routinization and incentives. A piece that is missing, however, is recognition that non-adherence and treatment interruption could be caused by factors beyond individual level factors such as motivation and present bias. The fully individual-level focus raises concerns about how plausible it would be for this approach to lead to sustained adherence among individuals who experience external threats to their ability to adhere, despite being motivated to do so and having routines.

1. Significance:

Strengths

- The need for innovation in the area of adherence support over time is a clear and compelling gap as this is a behavior that requires sustained maintenance over time.

Weaknesses

- While the investigators provide a compelling hypothesis regarding using incentives to establish routines early on, the exclusive focus on individual level determinants of non-adherence (present bias, motivation, forgetfulness) does not account for other social and structural determinants of adherence.

- The relevance of the work for other chronic conditions is mentioned in the aims and the proposal but this idea is not developed at all. It would be helpful to have more consideration of this as applicability to other chronic diseases would make this more significant.

2. Investigator(s):

Strengths

- The PI has extensive experience leading intervention studies in this setting and the proposed study represents an effort to extend that work. The team has worked together in the past and has demonstrated productivity. The roles of each investigator are well defined.
- The discussion of capacity building and career development is appreciated. It is notable that there is a named study coordinator, reflecting the strong existing research collaboration between the teams at RAND and Mildmay clinic.

Weaknesses

- none

3. Innovation:

Strengths

- There is innovation in the premise of the study, focused on testing the theory that anchoring and promoting the establishment of routines could promote sustained adherence.

Weaknesses

- The use of incentives itself is not innovative and the investigator team has already conducted extensive research in this area in this setting.

4. Approach:

Strengths

- Integration of qualitative and quantitative methods is appropriate for the study purpose and scope and will provide holistic information.
- Use of surveys at both 3 and 12 months will give some sense of sustained adherence. While the real gap is sustaining adherence consistently over longer periods, this will give some indication of the feasibility and effectiveness of this approach.
- Use of FGDs in Aim 3 is appropriate and could provide important data for understanding the intervention experience and processes.

Weaknesses

- Time allocated for ADAPT-ITT process (6 months) is short and may not facilitate meaningful engagement with the full range of steps and rich analysis of data. The investigators indicate having added more detail to Phase 1 description, but the current presentation is disproportionately focused on the *content of the interview guide*, with very little discussion of the steps in the ADAPT-ITT process.
- There is also disconnect between the Aim 1 analysis and the steps of the ADAPT-ITT process. Description of analysis stops at code reports and does not describe how they will be used to adapt the intervention.

- Who will conduct qualitative interviews? No detail provided on the “facilitators”.
- The investigators mention documenting both themes that are commonly discussed as well as those that are not in the aim 1 qual analysis. However, there is no discussion of how these data will be handled – less commonly discussed themes could be reflective of critical inductive findings that certain participants bring up despite not being a focus of the study guide and that the authors had not considered beforehand.
- For Aim 3, analysis of FGDs is described as the same as the approach for IDIs, which fails to account for the group dynamics, which can be a critical part of the production of data in FGDs. Again, how code reports will be interpreted and used to generate findings is not clear.

5. Environment:

Strengths

- The Mildmay clinic offers the potential study population and has an established history of collaboration.

Weaknesses

- none

Study Timeline:

Strengths

- None noted by reviewer.

Weaknesses

- Adapt component condenses many steps into a very short time period with limited discussion of the details involved in this process.

Protections for Human Subjects:

Acceptable Risks and/or Adequate Protections

Data and Safety Monitoring Plan (Applicable for Clinical Trials Only):

Acceptable

Inclusion Plans:

- Sex/Gender: Distribution justified scientifically
- Race/Ethnicity: Distribution justified scientifically
- For NIH-Defined Phase III trials, Plans for valid design and analysis: Not applicable
- Inclusion/Exclusion Based on Age: Distribution justified scientifically

Vertebrate Animals:

Not Applicable (No Vertebrate Animals)

Biohazards:

Not Applicable (No Biohazards)

Resubmission:

- The investigators provided a detailed response and made critical revisions. The revisions to the phase 1 qualitative section place disproportionate emphasis on the initial in-depth interviews and not enough detail on the adaptation process.

Applications from Foreign Organizations:

- Mildmay and RAND have a longstanding collaboration that will facilitate the proposed study.

Resource Sharing Plans:

Acceptable

Budget and Period of Support:

Budget Modifications Recommended (in amount/time)

Recommended budget modifications or possible overlap identified:

- It is noted that travel to Uganda does not appear to be included in the budget.

CRITIQUE 2

Significance: 3
Investigator(s): 1
Innovation: 2
Approach: 3
Environment: 1

Overall Impact: This application will develop, pilot test, and refine an intervention to facilitate pill-taking routines to improve HIV treatment adherence among PLHIV in Kampala, Uganda. Interventions to promote habit development are essential for adherence to ART regimes and adherence to other health behaviors. The hypothesis that providing incentives for taking the medication in combination with established routines (rather than providing incentives for just taking the medication) may help to consolidate pill-taking habits, is innovative. The approach involves 4 well-developed phases including formative interviews, systematic intervention adaptation process, pilot trial of the adapted intervention, and focus groups to assess acceptability and implementation issues. Overall, this is very good application. The scientific premise is clear and decisions are well-thought and justified. The intervention will not certainly address all factors that disrupt adherence (e.g., mental health, substance use, homelessness). However, this proof-of-concept study may result in a scalable intervention to promote adherence in low resource settings, in addition to providing further information about developing healthy habits. Remaining concerns are the assessment of adherence by averaging 9 months of MEMS cap data and questions about the significance of the preliminary data in Figure 2.

1. Significance:

Strengths

- High ART adherence is needed to achieve viral suppression and control the HIV epidemic.
- Understanding adherence behaviors is significant for multiple medical conditions.
- Simple, low-cost, scalable interventions can have significant public health impact.
- Integration of the intervention within the clinic procedures can help identify implementation challenges and opportunities, which will facilitate dissemination and scalability.
- The intervention can easily be adapted to other clinical and nonclinical settings.

Weaknesses

- Individuals who experience psychosocial and structural barriers to adherence are less likely to sustain other habits, comply with healthcare visits, follow instructions (such as bringing the MEMs cap to clinic visits), and remain in care.
- Figure 2 suggests that, among participants who did not associate pill-taking with other routines, the effect of removing incentives on adherence was only 3%.

2. Investigator(s):

Strengths

- The PI has expertise in behavioral economics which provides the theoretical underpinning for the proposed intervention.
- The PI has 10 years of experience working in Uganda and with the Mildmay Clinic.

Weaknesses

- Unclear if the team has expertise in longitudinal and time series analyses.

3. Innovation:

Strengths

- Linking pill-taking to other routines is the main innovation of the study.
- The combination of developing personalized action plans, reminder messages with information, and incentives is innovative.
- Using reminder messages that include procedural information is innovative as is assessing routinization through MEMs cap**.**

Weaknesses

- Incentives and reminder messaging are not novel interventions

4. Approach:

Strengths

- A strong conceptual framework guides all aspects of the design and intervention.
- The sequence of formative work, ADAPT-ITT, feasibility/acceptance/efficacy pilot trial, and qualitative interviews to address implementation issues is a strength.
- Using MEMS cap to measure routinization is a strength.
- Using multiple complementary measures of adherence (MEMS cap, self-report, viral load) is also a strength.

Weaknesses

- There are remaining concerns about how MEMS cap data will be analyzed and about using 9-month average measures of adherence, as this will not capture changes or trends following the intervention.
- Collecting process information may be beneficial to assess acceptability and adapt the intervention, for example, to determine whether participants become desensitized to the text messages or whether they read them and remember the content.
- As the time needed for habit development has not been studied in health settings, following more closely pill-taking routinization can provide information about how habits are consolidated.

5. Environment:

Strengths

- The research support and resources at the RAND Corporation are excellent.
- Investigators have collaborated with the Mildmay Clinic in Uganda in numerous studies.

Weaknesses

- None noted

Study Timeline:

Strengths

- The timeline seems adequate to complete the study aims.

Weaknesses

- None noted

Protections for Human Subjects:

Acceptable Risks and/or Adequate Protections

- Adequate protections

Data and Safety Monitoring Plan (Applicable for Clinical Trials Only):

Acceptable

- The DSMP is adequate to anticipate and address safety issues.

Inclusion Plans:

- Sex/Gender: Distribution justified scientifically
- Race/Ethnicity: Distribution justified scientifically
- For NIH-Defined Phase III trials, Plans for valid design and analysis: Not applicable
- Inclusion/Exclusion Based on Age: Distribution justified scientifically
- Includes about 70% women, reflecting the demographics of the clinic. Children under the age of 18 will be excluded because the clinic focuses on adult care

Vertebrate Animals:

Not Applicable (No Vertebrate Animals)

Biohazards:

Not Applicable (No Biohazards)

Resubmission:

- Generally responsive to critiques. Some of the responses are clarifications rather than changes. The 9-month average measure of adherence remains a concern.

Resource Sharing Plans:

Acceptable

Budget and Period of Support:

Recommend as Requested

CRITIQUE 3

Significance: 3
Investigator(s): 3
Innovation: 4
Approach: 4
Environment: 2

Overall Impact: This application proposes to develop a medication adherence intervention in collaboration with a Ugandan HIV clinic. The intervention, named INMIND, proposes to use small incentives and daily text messages to support treatment initiators anchoring ART pill-taking to an existing routine with the goal of establishing persistent ART adherence. The investigators propose 3 phases including a formative phase that will evaluate the feasibility and acceptability of INMIND, an intervention phase that will test for preliminary effectiveness (actually efficacy), and an adaptation phase based on that will inform a subsequent R01 larger study. The proposal is largely responsive to previous critique and has the potential to be successful. Enthusiasm for the proposal was limited by having many Approach concerns including that there was a lack of certainty about who the target population will be.

1. Significance:

Strengths

- ART medication adherence is a significant public health problem.
- The intervention is in reaction to a common reason for non-adherence – forgetting.
- Supporting/influencing patients when they initiate medication taking might influence long term behavior.

Weaknesses

- As the intervention relies on having funds for rewards, and many clinics may struggle with having discretionary money, it is not clear how scalable this approach could be. However, this is relatively low cost.

2. Investigator(s):

Strengths

- The PI has worked with this clinic on several NIH projects.
- Many experienced investigators with background in behavioral economics.

Weaknesses

- A prior critique stated, “The administrative contribution of Mukasa (8-10%) appears to be clear. However, her scientific contribution is unclear. She would be more suited for a Project Manager or Project Coordinator roles rather than as a Co-Investigator.” However, as per her biosketch, she says, “I will support the research team to provide administrative and programmatic oversight for implementation of the proposed research.” This critique is not addressed as she is still a co-I without her providing a statement of her scientific contribution.

3. Innovation:

Strengths

- Intervening specifically at initiation is innovative.
- A novel measure of behavior routinization is proposed.

Weaknesses

- Targeting forgetfulness is not an innovative strategy.

4. Approach:

Strengths

- Theory-based approach.
- The approach is appropriate for an R34 (formative work, test with pilot, refine intervention).
- The involvement of the community advisory board is a strength.
- The application of the ADAPT-ITT framework looks appropriate.
- The intervention is performed for 3 months followed by 9 months of observation to evaluate behavioral persistence. The 9 months is a good period of time for an R34 to observe after intervention.
- Evidence was given that they can recruit the number of proposed initiators.
- The plan to prevent attrition is well described.

Weaknesses

- Small rewards may overcome lack of motivation and help establish adherence as a routine. But it is not clear that for medication adherence this will be sustainable. This proposal will test that for 9 months beyond the intervention but medication adherence is a high hurdle for those challenged with it.

- The proposal states that "existing anchoring interventions have not been successful at supporting routine creation during" the relatively long time of 3 months. So it sounds like they are modifying a failed approach with an incentive. If that is so, it seems it is going to have to be highly effective in order to make a difference.
- A "major concern" is to ensure participants are not denied prize drawings due to circumstances beyond their control. The investigators will "ask participants about such obstacles and find solutions with clinic staff" but it is not clear how they know they will be able to find solutions. Also, this would have been a good thing to explore when drafting this proposal given the ongoing relations the investigators have with this clinic rather than have it occur only after funded given that it is a major concern.
- The proposal appropriately states that "An important issue will be integrating the intervention in the routine clinic flow to avoid burdening either participants or providers and to confirm the feasibility of intervention procedures." However what evidence is there that this is likely not to burden them and thus be a problem with feasibility and sustainability?
- The population for this study has not been determined. The proposal says they will launch it in the HIV clinic population but then also says "We will discuss with the medical staff and Mildmay's CAB whether to limit recruitment to young, male clients with less than primary education. If they are considering a targeted subpopulation, there should be a background section about this earlier in the grant. It also seems odd to say that the population for the study may be this or that rather than a clear approach. Additionally, why were these discussions not had in the preparation of the grant to make the decision? Additionally, it seems the greatest impact is expected in this small group but it is not clear from the grant how small is this group.
- The proposal states that they will "assess program dynamics over the 12-month study period" but it was not clear what this meant or how it would be done.
- At recruitment, participants are given an explanation and a one page leaflet laying out the strategy for anchoring to an existing routine. However, they stated that they might target young males with less than primary education. So it is not clear how they would be able to read even a simple language leaflet.
- Again, with text messages planned, if they are targeting young males who have little to no education, what evidence is there that they read and use text messages.
- Mention was made in the message group that they will receive daily text messages reminding them of their personalized action plans. However, it was not clear where or how these "personalized action plans" were created.
- Participants are asked to get an extra viral load test if there is none routinely collected within three months of the baseline visit or the month 12 study visit. It was not clear if they provide an incentive for this or just expect the participant to do it when told.
- The analysis should explore if young low education males are more likely to have present bias.
- The description of the post-intervention focus groups was only one paragraph and used vague language rather than described methods: "We will also talk with clinic staff to gauge their perception of intervention feasibility, limitations, and sustainability…"
- It was surprising to see a limitations section that describes no expected limitations.
- Minor - In response to a critique, the investigators increased the incentive frequency to monthly rather than only once after three months. While this may increase the efficacy, it also modestly decreases the feasibility by increasing the cost.

5. Environment:

Strengths

- RAND Corporation is an excellent environment for research.
- There is a successful track record of working with Mildmay Clinic in Uganda.

Weaknesses

- None

Study Timeline:

Strengths

- The timeline seems adequate.

Weaknesses

- None noted.

Protections for Human Subjects:

Acceptable Risks and/or Adequate Protections

Data and Safety Monitoring Plan (Applicable for Clinical Trials Only):

Acceptable

Inclusion Plans:

- Sex/Gender: Distribution not justified scientifically
- Race/Ethnicity: Distribution justified scientifically
- For NIH-Defined Phase III trials, Plans for valid design and analysis: Not applicable
- Inclusion/Exclusion Based on Age: Distribution not justified scientifically
- If they are going to select only young males as they said they might, they did not adequately justify that.

Vertebrate Animals:

Not Applicable (No Vertebrate Animals)

Biohazards:

Not Applicable (No Biohazards)

Resubmission:

- The resubmission is mostly responsive. The issue noted above is whether or not Dr. Mukasa contributes scientifically.

Resource Sharing Plans:

Acceptable

Budget and Period of Support:
Recommend as Requested

THE FOLLOWING SECTIONS WERE PREPARED BY THE SCIENTIFIC REVIEW OFFICER TO SUMMARIZE THE OUTCOME OF DISCUSSIONS OF THE REVIEW COMMITTEE, OR REVIEWERS' WRITTEN CRITIQUES, ON THE FOLLOWING ISSUES:

PROTECTION OF HUMAN SUBJECTS: ACCEPTABLE

INCLUSION OF WOMEN PLAN: ACCEPTABLE

INCLUSION OF MINORITIES PLAN: ACCEPTABLE

INCLUSION ACROSS THE LIFESPAN: ACCEPTABLE

COMMITTEE BUDGET RECOMMENDATIONS: The budget was recommended as requested.

Footnotes for 1 R34 MH122331-01A1; PI Name: Linnemayr, Sebastian

+ Derived from the range of percentile values calculated for the study section that reviewed this application.

NIH has modified its policy regarding the receipt of resubmissions (amended applications).See Guide Notice NOT-OD-18-197 at https://grants.nih.gov/grants/guide/notice-files/NOT-OD-18-197.html. The impact/priority score is calculated after discussion of an application by averaging the overall scores (1-9) given by all voting reviewers on the committee and multiplying by 10. The criterion scores are submitted prior to the meeting by the individual reviewers assigned to an application, and are not discussed specifically at the review meeting or calculated into the overall impact score. Some applications also receive a percentile ranking. For details on the review process, see http://grants.nih.gov/grants/peer_review_process.htm#scoring.

MEETING ROSTER

HIV/AIDS Intra- and Inter-personal Determinants and Behavioral Interventions Study Section
AIDS and Related Research Integrated Review Group
CENTER FOR SCIENTIFIC REVIEW
HIBI
03/12/2020 - 03/13/2020

Notice of NIH Policy to All Applicants: Meeting rosters are provided for information purposes only. Applicant investigators and institutional officials must not communicate directly with study section members about an application before or after the review. Failure to observe this policy will create a serious breach of integrity in the peer review process, and may lead to actions outlined in NOT-OD-14-073 at https://grants.nih.gov/grants/guide/notice-files/NOT-OD-14-073.html and NOT-OD-15-106 at https://grants.nih.gov/grants/guide/notice-files/NOT-OD-15-106.html, including removal of the application from immediate review.

CHAIRPERSON(S)

STEPHENSON, ROB B, PHD
PROFESSOR
DEPARTMENT OF HEALTH BEHAVIOR
AND BIOLOGICAL SCIENCES
SCHOOL OF NURSING
UNIVERSITY OF MICHIGAN
ANN ARBOR, MI 48109

MEMBERS

BARRINGTON, CLARE L, MPH, PHD *
ASSOCIATE PROFESSOR
DEPARTMENT OF HEALTH BEHAVIOR
GILLINGS SCHOOL OF GLOBAL PUBLIC HEALTH
UNIVERSITY OF NORTH CAROLINA
CHAPEL HILL, NC 27599

BUTLER, LISA MICHELLE, MPH, PHD
ASSOCIATE RESEARCH PROFESSOR
INSTITUTE FOR COLLABORATION ON HEALTH,
INTERVENTION, AND POLICY
UNIVERSITY OF CONNECTICUT
STORRS, CT 06269

CHEN, XINGUANG, MD, PHD, MPH *
PROFESSOR
DEPARTMENT OF EPIDEMIOLOGY
COLLEGE OF PUBLIC HEALTH AND HEALTH PROFESSIONS
UNIVERSITY OF FLORIDA
GAINSVILLE, FL 32610

CORSI, KAREN F, SCD
ASSOCIATE PROFESSOR
DEPARTMENT OF PSYCHIATRY
SCHOOL OF MEDICINE
UNIVERSITY OF COLORADO, DENVER
DENVER, CO 80206

DWORKIN, MARK S, MD, MPH *
PROFESSOR
DIVISION OF EPIDEMIOLOGY AND BIOSTATISTICS
SCHOOL OF PUBLIC HEALTH
UNIVERSITY OF ILLINOIS AT CHICAGO
CHICAGO, IL 60612

DYER, TYPHANYE V, MPH, PHD *
ASSISTANT PROFESSOR
DEPARTMENT OF EPIDEMIOLOGY
SCHOOL OF PUBLIC HEALTH
UNIVERSITY OF MARYLAND
COLLEGE PARK, MD 20742

GLASMAN, LAURA R, PHD *
ASSISTANT PROFESSOR
DEPARTMENT OF PSYCHIATRY AND BEHAVIORAL MEDICINE
CENTER FOR AIDS INTERVENTION
MEDICAL COLLEGE OF WISCONSIN
MILWAUKEE, WI 53223

GRAHAM, SUSAN MARIE, MD, MPH, PHD
ASSOCIATE PROFESSOR
DIVISION OF ALLERGY AND INFECTIOUS DISEASES
DEPARTMENTS OF MEDICINE AND GLOBAL HEALTH
SCHOOL OF MEDICINE
UNIVERSITY OF WASHINGTON
SEATTLE, WA 98104

GWADZ, MARYA, PHD
PROFESSOR
ASSOCIATE DEAN FOR RESEARCH
SILVER SCHOOL OF SOCIAL WORK
NEW YORK UNIVERSITY
NEW YORK, NY 10003

HANSEN, NATHAN B, PHD
DEPARTMENT HEAD AND PROFESSOR
DEPARTMENT OF HEALTH PROMOTION AND BEHAVIOR
COLLEGE OF PUBLIC HEALTH
UNIVERSITY OF GEORGIA
ATHENS, GA 30602

HOFFMAN, SUSIE, DRPH *
RESEARCH SCIENTIST
HIV CENTER FOR CLINICAL AND BEHAVIORAL STUDIES
NEW YORK STATE PSYCHIATRIC INSTITUTE
NEW YORK, NY 10032

HORVATH, KEITH JOSEPH, PHD
ASSOCIATE PROFESSOR
DEPARTMENT OF CLINICAL PSYCHOLOGY
SAN DIEGO STATE UNIVERSITY
SAN DIEGO, CA 92120

IWELUNMOR, JULIET, PHD *
ASSOCIATE PROFESSOR
DEPARTMENT OF BEHAVIORAL SCIENCE AND
HEATLH EDUCATION
COLLEGE FOR PUBLIC HEALTH AND SOCIAL JUSTICE
SAINT LOUIS UNIVERSITY
ST. LOUIS, MO 63104

KIPKE, MICHELE D, PHD
PROFESSOR
DEPARTMENT OF PEDIATRICS AND PREVENTIVE MEDICINE
KECK SCHOOL OF MEDICINE
UNIVERSITY OF SOUTHERN CALIFORNIA
LOS ANGELES, CA 90027

LEHMAN, WAYNE E.K., PHD *
SENIOR RESEARCH SCIENTIST
INSTITUTE OF BEHAVIORAL RESEARCH
TEXAS CHRISTIAN UNIVERSITY
FORT WORTH, TX 76129

LIPPMAN, SHERI ANN, PHD *
ASSOCIATE PROFESSOR
CENTER FOR AIDS PREVENTION STUDIES
SCHOOL OF MEDICINE
UNIVERSITY OF CALIFORNIA, SAN FRANCISCO
SAN FRANCISCO, CA 94143

LOVEJOY, TRAVIS IAN, MPH, PHD *
ASSOCIATE PROFESSOR
DEPARTMENT OF PSYCHIATRY
SCHOOL OF MEDICINE
OREGON HEALTH AND SCIENCE UNIVERSITY
PORTLAND, OR 97239

MERCHANT, ROLAND C, MD, MPH, SCD
PROFESSOR
DEPARTMENT OF EMERGENCY MEDICINE
BRIGHAM AND WOMEN'S HOSPITAL
BOSTON, MA 02115

MONTGOMERY, ELIZABETH TRASK, PHD *
SENIOR RESEARCH EPIDEMIOLOGIST
WOMEN'S GLOBAL HEALTH IMPERATIVE
RESEARCH TRAINGLE INSTITUTE
SAN FRANCISCO, CA 94104--242

PENCE, BRIAN W., PHD, MPH *
ASSOCIATE PROFESSOR
DEPARTMENT OF EPIDEMOLOGY
GILLINGS SCHOOL OF GLOBAL PUBLIC HEALTH
UNIVERSITY OF NORTH CAROLINA
CHAPEL HILL, NC 27516

RAMSEY, SUSAN E, PHD
ASSOCIATE PROFESSOR
DIVISION OF GENERAL INTERNAL MEDICINE
RHODE ISLAND HOSPITAL
BROWN UNIVERSITY
PROVIDENCE, RI 02903

SANTOS, GLENN-MILO, MPH, PHD *
SENIOR RESEARCH SCIENTIST
COMMUNITY HEALTH SYSTEMS
SCHOOL OF NURSING
UNIVERSITY OF CALIFORNIA, SAN FRANCISCO
SAN FRANCISCO, CA 94102

SHEEHAN, DIANA MONTSERRAT, PHD, MPH *
ASSISTANT PROFESSOR
DEPARTMENT OF EPIDEMIOLOGY
COLLEGE OF PUBLIC HEALTH AND SOCIAL WORK
FLORIDA INTERNATIONAL UNIVERSITY
MIAMI, FL 33199

SSEWAMALA, FRED M, PHD
PROFESSOR
INSTITUTE FOR PUBLIC HEALTH
BROWN SCHOOL
WASHINGTON UNIVERSITY
ST. LOUIS, MO 63130

STOCKMAN, JAMILA KINSHASA, MPH, PHD
ASSOCIATE PROFESSOR
DIVISION OF GLOBAL PUBLIC HEALTH
DEPARTMENT OF MEDICINE
SCHOOL OF MEDICINE
UNIVERSITY OF CALIFORNIA, SAN DIEGO
LA JOLLA, CA 92093

SULLIVAN, PATRICK SEAN, DVM, PHD
PROFESSOR
DEPARTMENT OF EPIDEMIOLOGY
ROLLINS SCHOOL OF PUBLIC HEALTH
EMORY UNIVERSITY
ATLANTA, GA 30322

SULLIVAN, TAMI P, PHD *
ASSOCIATE PROFESSOR
DEPARTMENT OF PSYCHIATRY
SCHOOL OF MEDICINE
YALE UNIVERSITY
NEW HAVEN, CT 06511

THAMES, APRIL D, PHD *
ASSOCIATE PROFESSOR
DEPARTMENT OF PSYCHOLOGY
UNIVERSITY OF SOUTHERN CALIFORNIA
LOS ANGELES, CA 90089

TOBIN, KARIN E, PHD
ASSOCIATE PROFESSOR
DEPARTMENT OF HEALTH, BEHAVIOR, AND SOCIETY
BLOOMBERG SCHOOL OF PUBLIC HEALTH
JOHNS HOPKINS UNIVERSITY
BALTIMORE, MD 21205

TURAN, JANET M, MPH, PHD
PROFESSOR
DEPARTMENT OF HEALTH CARE ORGANIZATION
AND POLICY
SCHOOL OF PUBLIC HEALTH
UNIVERSITY OF ALABAMA AT BIRMINGHAM
BIRMINGHAM, AL 35294

WANG, BO, PHD *
ASSOCIATE PROFESSOR
POPULATION AND QUANTITATIVE HEALTH SCIENCES
UNIVERSITY OF MASSACHUSETTS MEDICAL SCHOOL
WORCESTER, MA 01605

WILSON, TRACEY ELIZABETH, PHD *
PROFESSOR
DEPARTMENT OF COMMUNITY HEALTH SCIENCES
SCHOOL OF PUBLIC HEALTH
DOWNSTATE MEDICAL CENTER
THE STATE UNIVERSITY OF NEW YORK
BROOKLYN, NY 11203

WILTON, LEO, PHD, MPH *
PROFESSOR
DEPARTMENT OF HUMAN DEVELOPMENT
COLLEGE OF COMMUNITY AND PUBLIC AFFAIRS
BINGHAMTON UNIVERSITY
BINGHAMTON, NY 13902

SCIENTIFIC REVIEW OFFICER

RUBERT, MARK P, PHD
SCIENTIFIC REVIEW OFFICER
CENTER FOR SCIENTIFIC REVIEW
NATIONAL INSTITUTES OF HEALTH
BETHESDA, MD 20892

EXTRAMURAL SUPPORT ASSISTANT

IM, CHRISTINE
ADMINISTRATIVE ASSISTANT
CENTER FOR SCIENTIFIC REVIEW
NATIONAL INSTITUTES OF HEALTH
BETHESDA, MD 20892

* Temporary Member. For grant applications, temporary members may participate in the entire meeting or may review only selected applications as needed.

Consultants are required to absent themselves from the room during the review of any application if their presence would constitute or appear to constitute a conflict of interest.
